# Genomic and expression analysis of glycosyl hydrolase family 35 genes from rice (*Oryza sativa *L.)

**DOI:** 10.1186/1471-2229-8-84

**Published:** 2008-07-30

**Authors:** Waraporn Tanthanuch, Mallika Chantarangsee, Janjira Maneesan, James Ketudat-Cairns

**Affiliations:** 1 National Synchrotron Research Center, 111 University Avenue, Nakhon Ratchasima, 30000, Thailand; 2 School of Biochemistry, Institute of Sciences, Suranaree University of Technology, Nakhon Ratchasima, 30000, Thailand

## Abstract

**Background:**

Many plant β-galactosidases (Bgals) have been well characterized and their deduced biological functions mainly involve degradation of structural pectins, xyloglucans or arabinogalactoproteins in plant cell walls. However, gene multiplicity in glycosyl hydrolase family 35 (GH35), to which these proteins belong, implies diverse functions. In this study, the gene multiplicity, apparent evolutionary relationships and transcript expression of rice Bgal genes were examined, in order to predict their biological functions.

**Results:**

Fifteen rice Bgal genes were identified in the plant genome, one of which encodes a protein similar to animal Bgals (*OsBgal9*), and the remaining 14 fall in a nearly plant-specific subfamily of Bgals. The presence of both classes of Bgals in bryophytes, as well as vascular plants, suggests both gene lineages were present early in plant evolution. All 15 proteins were predicted to contain secretory signal sequences, suggesting they have secretory pathway or external roles. RT-PCR and database analysis found two distinct lineages to be expressed nearly exclusively in reproductive tissues and to be closely related to *Arabidopsis *Bgals expressed most highly in flower and pollen. On the other hand, *OsBgal6 *is expressed primarily in young vegetative tissues, and alternative splicing in panicle prevents its production of full-length protein in this reproductive tissue. *OsBgal11 *also showed alternative splicing to produce different length proteins. OsBgal13 produced by recombinant expression in *Escherichia coli *hydrolyzed α-L-arabinoside in addition to β-D-galactoside and β-(1→3)-, β-(1→4)- and β-(1→6)- linked galacto-oligosaccharides.

**Conclusion:**

Rice *GH35 *contains fifteen genes with a diversity of protein sequences, predicted locations and expression and splicing patterns that suggest that OsBgals enzymes may play a variety of roles in metabolism of cell wall polysaccharides, glycoproteins and glycolipids.

## Background

β-Galactosidases (Bgals) (EC 3.2.1.23) catalyze the hydrolysis of the glycosidic bonds of terminal non-reducing β-D-galactosyl residues of oligosaccharides and β-D-galactopyranosides. This group of enzymes has a broad distribution, which encompasses plants, animals and microorganisms. The biological functions of these enzymes include degradation of structural polysaccharides in plant cell walls [1,2], thereby acting to control fruit softening during ripening [3], hydrolysis of dietary lactose [4,5] and degradation of glycolipids and proteoglycans in mammals [6,7], and metabolism of lactose and other galactosides in microorganisms [8,9]. The extensive diversity of Bgals raises the question of what is the basis of their different substrate specificities, which help determine their functions.

Glycosyl hydrolases (GH) have been grouped and classified as families on the basis of structural similarity [10] and Bgals fall into glycosyl hydrolase (GH) families 1, 2, 35, and 42, which are part of superfamily A (or Clan A). Based on amino acid sequence similarities [11], plant Bgals that have been described belong to GH family 35 (GH35). Like other glycosidase families, GH35 includes multiple genes in various plant species, including *Arabidopsis *[12], tomato [13], papaya [14], apple [15], *Vigna unguiculata *[16] and barley [17], suggesting that GH35 gene multiplicity is ubiquitous in plants.

Analysis of plant Bgal activities led to the proposal of two classes, I and II [18]. Class I is made up of enzymes with well characterized exo-β-(1→4)-galactanase activities that can specifically act on pectic β-(1→4)-galactan. Class II has hydrolytic activity on the β-(1→3)- and β-(1→6)-galactosyl linkages of arabinogalactan proteins (AGPs), but lacks activity toward pectic β-(1→4)-galactan [19,20], as reported in spinach leaves [20] and hypocotyls and young leaves of radish [18]. Class I OsBgals can specifically act on β-(1→4)-galactosyl residues in pectin and xyloglucan. Therefore, they could play an important role in modification of the architecture of the cell wall and intercellular attachment [1,2]. The apparent involvement of Bgals in pectin disassembly during fruit ripening has been reported in various plant species, including tomato [3,21,22], muskmelon [23], kiwifruit [24], persimmon [25], sweet cherry [26], mango [27], and peach [25]. Bgals expression also accompanies many stages of plant development in other tissues, for example, spinach leaves [20], mungbean seedlings [29], radish hypocotyls and young leaves [18], and meristem zones of roots, cotyledons, vascular tissues, trichomes, and pollen of tobacco [30,31]. Bgals that appear to catalyze galactose removal from xyloglucans during their disassembly were observed in cotyledons of nasturtium (*Tropueolum mujus *L.) seed [32], *Copaifera langsdorffii *seed [33], and *Hymenaea courbaril *seed, where they were reported to act in cooperation with α-xylosidase, β-glucosidase, and other enzymes to achieve xyloglucan degradation [34,35]. Therefore, Bgals appear to play a role in cell wall remodeling in many plant processes.

In rice, GH35 has not yet been well characterized, and the biological function of rice Bgals remain mysterious. Konno and Tsumuki [36] identified both soluble and cell-wall-bound Bgal, of which a 42 kDa soluble Bgal was purified and shown to release galactose from larch wood and rice cell wall arabinogalactans. Likewise, Kaneko and Kobayashi [37] isolated a Bgal with 40 and 47 kDa subunits from the medium of rice suspension cells. Recently, Chantarangsee et al. [38] characterized two recombinant Bgal isozymes, including the 90 kDa OsBgal1 and 72 kDa OsBgal2, which had different expression patterns, though both are found throughout plant growth. The complete rice genome sequence allows extensive study of rice GH35, so the gene structures, encoded protein sequences and phylogenetic relationships with other Bgals from rice and other organisms, which may provide clues to their evolution and possible functions, were determined. In addition, the transcript expression patterns of all rice GH35 *Bgals *have been determined and OsBgal13 was expressed in *Escherichia coli *to gain a clue to OsBgal physiological functions.

## Results and Discussion

### Identification of rice GH35 genes and their protein products

A total of 15 GH35 genes (defined *OsBgal*) were identified in rice genome databases (Table 1), each of which encodes a protein that contains the GH35 β-galactosidase active site consensus sequence G-G-P- [LIVM]-x-Q-x-E-N-E- [FY] [39]. Most of the encoded proteins contain a GH35 domain at the N-terminus and a Gal-lectin-like domain at the C-terminus. Although the speculated carbohydrate-binding function of these Gal-lectin domains is not yet proven, their existence and conserved nature has led to the suggestion that they may increase the catalytic efficiency on polysaccharide substrates [12]. The proteins from *OsBgal*2, *OsBgal*7 and *OsBgal9 *lack the Gal-lectin-like domain, suggesting that it is not necessary for the functions of these rice GH35 members.

**Table 1:** Summary of identified genes homologous to glycosyl hydrolase family 35 galactosidase

Gene name	BGI entries (AAAA...)^a^	Genomic Contig.	Chr.^b^	UniGene	Gene Locus (NCBI, RGP)	Gene Locus (TIGR)	Corresponding cDNA^c^	Number^d^		Tissue librarys^e^	Expression profile (TPM) Transcript per millon^f^								GEO profile^g^
											
								mRNA	ESTs		Cl.	Fw.	Lf.	P	Rt.	Sd.	St.	Vm	
*OsBgal1*	1002951	AP008209.1	3	Os.13559	Os03g0165400	LOC_Os03g06940	AF499737	5	99	Fw, Cl, P, Mx, Wp, UnT, Vm, Sd, St, Rt	121	241	46	120	14	92	23	217	Up: CKl, AB&GBd, AnX, Dw: AB&GB, NaCl, Na_3_AsO_4_, CKrl
*OsBgal2*	1003471	AP004729.3, AP008212.1	6	Os.87715	Os06g0573600	LOC_Os06g37560	AK102756	4	201	P, Lf, Mx, St, Rt, Cl, Fw, Wp, UnT	66	80	291	391	205	0	118	0	Up: CKl, AB&GBd, NaCl Dw: AnX, AB&GB, Na_3_AsO_4_, CKrl
*OsBgal3*	01012445, 01022298	AP008207.1, AP003546.4	1	Os.18562	Os01g0580200	LOC_Os01g39830	AK103045	2	44	Cl, Wp, P, St, Fw, Mx, UnT, Vm	115	43	0	37	0	30	47	217	Up: CKl, AB&GBd, AB&GB, Na_3_AsO_4_, NaCl Dw: AnX, CKrl
*OsBgal4*	ND	AP003297.3, AP008207.1	1	Os.9892	Os01g0875500	LOC_Os01g65460	AK101399	3	107	Cl, Fw, Mx, P, Rt, UnT, St, Wp	279	263	0	45	29	30	0	0	Up: CKl, AnX, AB&GBd, Na3AsO4, NaCl Dw: AB&GB
*OsBgal5*	1000473.1	AP003447.2, AP008207.1, AP003445.2	1	Os.41043	Os01g0533400	LOC_Os01g34920	AK119447	2	3	Mx, St	ND	ND	ND	ND	ND	ND	ND	ND	Up: Na_3_AsO_4_, CKrl Dw: CKl, AnX, NaCl
*OsBgal6*	101013714.1	AC135419.2, AC135429.2	5	Os.82841	P0636F09.15	LOC_Os05g35360	EU602310	ND	1	UnT	ND	ND	ND	ND	ND	ND	ND	ND	-
*OsBgal7*	01011567, 01017443	AP008208.1	2	Os.14358	Os02g0219200	LOC_Os02g12730	AK059059	7	57	P, Mx, St, Cl, UnT, Fw, Wp, Rt	24	21	0	135	14	92	78	0	Up: AnX, CKl, AB&GB, AB&GBd, NaCl, Na3AsO4 Dw: CKrl
*OsBgal8*	1001024	AP008209.1, DP000009.2	3	Os.22360	Os03g0255100	LOC_Os03g15020	AK067479	3	253	Fw, Mx, St, Lf, P, Rt, Cl, UnT, Sd, Wp	42	761	133	120	190	216	228	0	Up: AnX, CKl, AB&GBd, NaCl Dw: Na_3_AsO_4_
*OsBgal9*	ND	AP008211.1	5	Os.14570	Os05g0539400	LOC_Os05g46200	AK068572	2	66	Lf, St, P, Mx, Cl, UnT, Wp, Sd, Rt	12	0	122	52	14	247	110	0	Up: CKl, AB&GBd, AB&GB, NaCl Dw: AnX, Na_3_AsO_4_
*OsBgal10*	1005991	AP008214.1, AP003912.3	8	Os.18310	Os08g0549200	LOC_Os08g43570	AK069066	3	137	Mx, Fw, P, St, UnT	0	249	0	158	0	0	0	0	Up: AB&GBd, AB&GB, AnX Dw: Na_3_AsO_4_, CKrl
*OsBgal11*	1013958	AP008215.1	9	Os.49945	Os09g0539200	LOC_Os09g36810	EU603286	2	7	Mx, Sd	0	0	0	0	0	61	0	0	Up: AnX, Na_3_AsO_4_, NaCl Dw: CKl
*OsBgal12*	10005757.1	AP008216.1, DP000086.1	10	Os.46702	Os10g0330600	LOC_Os10g18400	AK119350	3	3	Mx, Fw	0	7	0	0	0	0	0	0	Up: AnX
*OsBgal13*	ND	AP008218.1	12	Os.52193	Os12g0429200	LOC_Os12g24170	AK065546	2	36	Mx, St, Lf, Fw, UnT, P, Wp, Cl	6	21	34	15	0	0	55	0	Up: CKl, AB&GBd, AB&GB Dw: AnX, Na_3_AsO_4_, NaCl
*OsBgal14*	10006090	ND	10	Os.22528	J090043H02	LOC_Os10g19960	AK242960	2	58	Fw, P, UnT, Sd, Wp	0	358	0	7	0	61	0	0	-
*OsBgal15*	1003798.1	AP004733	6	NF	AP004733	LOC_Os06g42310	EU051629	ND		ND	ND	ND	ND	ND	ND	ND	ND	ND	-

^a^Contig number in Beijing Genome Institute (the numbers given are the Genbank Accession without the initial 'AAAA').

^b^Chromosome locations from mapping of the corresponding genes on the 12 rice chromosomes in GenBank.

^c^Accessions of full-length cDNA clones of japonica rice databases [64] or indica clones from this study (*OsBgal6*, *OsBgal11 *and *OsBgal15*).

^d^The number of mRNA and EST identified by UniGene from the NCBI Genbank database.

^e^Tissue libraries means the source tissue from which the corresponding ESTs were isolated. Tissue abbreviations are: Cl: callus, Fw: Flower, Lf: leaf, P: Panicle, Rt: root, Sd: Seed, St: Stem, Mx: Mixed Tissues, Vm: Vegetative meristem, Wp: whole plant, UnT: unspecified tissue.

^f^Expression profile means transcript frequency per million calculated from the number of ESTs in a certain library. ND: not determined.

^g^GEO (Gene Expression Omnibus public repository of microarray and other forms of high-throughput data submitted by the scientific community). Abbreviations are: Up: up-regulated, Dw:down-regulate, CKrl: Cytokinin effect on rice roots and leaves: time course, CKl: Cytokinin-inducible type-A response regulator OsRR6 overexpression effect on rice leaves, AB&GB: Abscisic acid and gibberellin effect on calluses, gene expression array-based, log ratio, AB&GBd: Abscisic acid and gibberellin effect on calluses (dye-swap), AnX: Anoxia effect on rice coleoptiles, NaCl: Salinity stress response, Na_3_AsO_4_: Sodium arsenate effect on Azucena and Bala varieties.

The deduced amino acid sequences of the OsBgal proteins were used to predict their putative signal peptides, protein lengths, molecular masses, pI values, possible N-glycosylation sites, and cellular destinations (Table 2). All OsBgals contain putative signal peptides, which range in length from 20 to 36 amino acids, except for that of OsBgal8 which is predicted to have 62 amino acids. The mature OsBgal proteins were predicted to contain from 653 (OsBgal9) to 894 (OsBgal8) amino acids, corresponding to molecular masses of 73.5 to 97.9 kDa. Most of the proteins were predicted to have pI in the acidic range (5.56–6.76), except those of OsBgal2, OsBgal7 and OsBgal10 are in the basic range (7.65–9.1) (Table 2). Only OsBgal2 and OsBgal7 lacked putative N-glycosylation sites, while the other enzymes were predicted to contain from 2 to 10 sites. Most OsBgal members are predicted to localize to the organelles of the secretory pathway, for instance, the Golgi apparatus, endoplasmic reticulum, or vacuole, or to be secreted. Only 3 OsBgals, including OsBgal9, OsBgal10 and OsBgal11, were predicted to be localized in the lysosome-like vacuole, which correlates to the location of mammalian β-galactosidases in the lysosome (Table 2). Although OsBgal10 and OsBgal11 are very similar in predicted mature protein length and possible cellular destinations, their predicted pI values are quite different. OsBgal5, OsBgal12, OsBgal14 and OsBgal15 all have high numbers of glycosylation sites, similar MW, pI and predicted possible destinations, which suggests that they may have redundant or overlapping functions.

**Table 2:** Predicted rice GH family 35 β-galactosidase protein properties and locations.

Gene Name	Gene Locus (NCBI, RGP)	Pre-protein			Mature Protein				
			
		MW^a ^(kDa)	AA^b^	Cleavage site^c^	MW^a^	AA^b^	pI^a^	N-gly site^d^	Possible destination^e^
*OsBgal1*	Os03g0165400	92.2	841	25–26	89.8	816	5.78	2	Gol, Vac, ER ext
*OsBgal2*	Os06g0573600	78.3	715	20–21	76.5	695	7.65	0	Gol, PM, IMP, MII, ER ext
*OsBgal3*	Os01g0580200	91.5	827	24–25	89.5	803	5.59	2	Gol, PM, IMP, MII, ER, Sec
*OsBgal4*	Os01g0875500	94.7	851	29–30	91.7	822	6.76	6	Gol, Cis-Gol, MII, ER, Sec
*OsBgal5*	Os01g0533400	91.7	827	25–26	88.9	802	5.66	8	Gol, MII, ER
*OsBgal6*	P0636F09.15	90.6	811	17–18	88.9	794	6.61	5	IMP, MC in, Sec, Cy, N
*OsBgal7*	Os02g0219200	78	729	36–37	76.8	693	8.71	0	Gol, MII, ER, Sec
*OsBgal8*	Os03g0255100	103.7	956	62–63	96.5	894	5.62	7	PM, IMP, ER, Sec
*OsBgal9*	Os05g0539400	75.7	673	20–21	73.5	653	5.57	6	Vac-Lys, IMP, MC in, Per, ERl, N, Sec, Cy
*OsBgal10*	Os08g0549200	94.7	848	23–24	92.8	825	9.1	5	Vac-Lys, PM, IMP, MC in, ER, Cy, Sec
*OsBgal11*	Os09g0539200	94.11	838	25–26	91.9	813	6.52	3	Vac-Lys, Gol, PM, IMP, MII, Mic, ER, MC in, Sec
*OsBgal12*	Os10g0330600	92	828	23–24	89.7	805	6.07	10	Gol, MII, ER, Sec
*OsBgal13*	Os12g0429200	101.0	919	31–32	97.9	888	5.56	5	IMP, ER, Sec
*OsBgal14*	J090043H02	92.2	828	28–29	89.6	800	5.99	9	PM, IMP, MII, ER, Sec
*OsBgal15*	AP004733	89.6	809	23–24	87.3	789	6.17	8	Gol, MII, ER, Sec

^a ^determined by ProtParam. ^b ^AA means number of amino acids. ^c^predicted by SignalP [35]. ^d^predicted by NetNGlyc at the Expasy proteomics server  and manually curated to remove NPS/T sites, ^e^cellular locations predicted by PSORT [65], abbreviated as PM: plasma membrane, MII: Type II membrane protein, Gol: Golgi, IMP: Integral Membrane Protein, Sec: Secreted, ER: Endoplasmic reticulum; ext: extracellular; l: lumen, MC: Mitochondrial; in: inner membrane, Cy: Cytoplasmic, N: nuclear, Vac: vacuoles, Vac-Lys: Lysosome-like vacuoles, Per: Peroxisomal, and Mic: microsomal.

### Phylogenetic analysis

**Figure 1:**
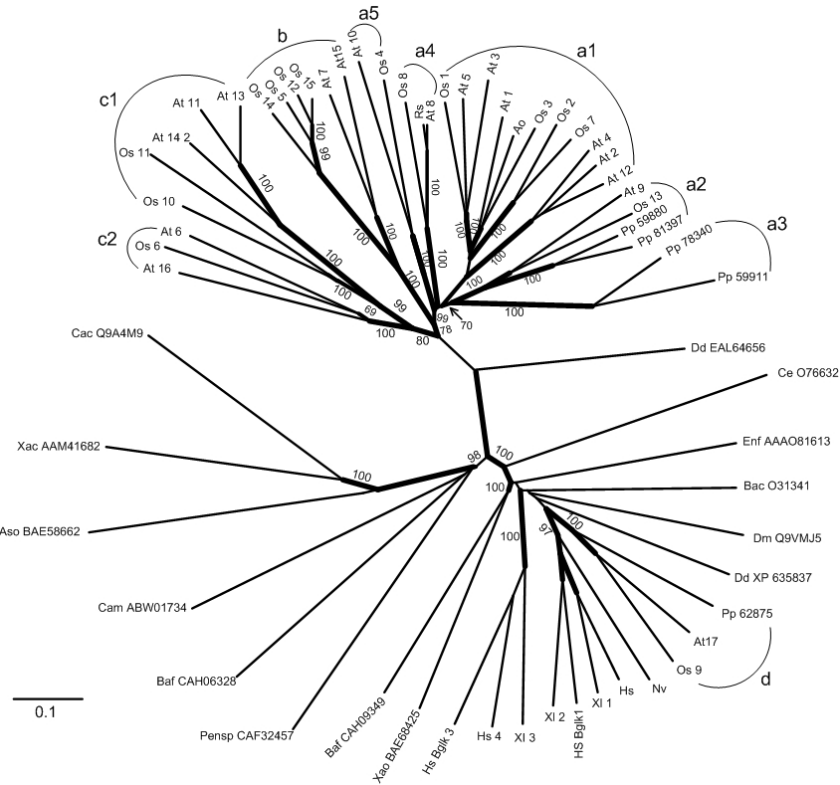
**Phylogenetic relationship among the rice and other glycosyl hydrolase family 35 proteins.** Deduced amino acid sequences were aligned using ClustalX program [63] and edited with Gendoc [62], then the tree were created using neighbor joining method and analyzed with 1000 bootstrap replicates, for which the percent reproducibility is shown on the branches that gave higher than 50% reproducibility. A tree was also made by maximum parsimony with the PHYLIP Protpars program, and branches supported by this analysis are shown with thicker lines. The rice sequences are named Os and a number according to the OsBgal numbers in Tables 1 and 2. *Arabidopsis thaliana *sequences are indicated as At, followed by the AtBGAL numbers of Ahn et al. [12]. Sequences derived from the genome of the bryophyte *Physcomitrella patens subsp. patens are given as Pp and the Genbank accession number without the initial "EDQ." Other β-galactosidase or related sequences (and their accession numbers) shown are: Ao, Asparagus officinalis *(CAA54525); Aso, *Aspergillus oryzae *(BAE58662); Bac, *Bacillus circulans *(O31341); Baf, *Bacteroides fragilis *(CAH09349 and CAH06328); Cac, *Caulobacter crescentus *(Q9A4M9); Cam, *Caldivirga_maquiingensis *(ABW01734); Ce, *Caenorhabditis elegans *(O76632); Dd (*Dictyostelium discoideum*) (EAL64656 and XP_635837); Dm, *Drosophila melanogaster *(Q9VMJ5); Enf, *Enterococcus faecalis *(AAO81613); Hs, human (*Homo sapiens) b-galactosidase *(P16278); HsBglk1, human b-galactosidase-1-like protein, (Q6UWU2); Hs Bglk 3, human b-galactosidase-1-like protein 3, (Q8NCI6); Hs_4, human locus 89944 (Q8IW92); Nv, sea anemone (*Nematostella vectensis*, XP_001631933); Pensp, *Penicillium *sp. (CAF32457); Rs, radish (*Raphanus sativus*) (BAD20774); Xac, *Xanthomonas campestris *(AAM41682); Xao, *Xanthomonas oryzae *(BAE68425); Xl African three-toed frog (*Xenopus laevis*): Xl 1 (AAI24928), Xl 2 (AAH74351), Xl 3 (AAH46858).

The multiple alignment of full-length protein sequences was used to construct an unrooted phylogenetic tree. The phylogenetic tree, which includes all the GH35 genes identified in the rice (monocot), *A. thaliana *(dicot) and *Physcomitrella patens *(a bryophyte moss) genomes, as well as representatives of animals, fungi, protists, archaea, and eubacteria, has three major branches, one of which is nearly plant specific (except for one *Dictyostelium discoideum *gene product), one of which contains representatives of animals, eubacteria and plants, and one of which contains archaeal, eubacterial and fungal enzymes (Figure 1). Almost all the rice, *A. thaliana*, and *P. patens *enzymes fall within the plant-specific cluster, except *OsBgal9*, *AtBGAL*17 and *P. patens *EDQ62875, which fall in the cluster with animal β-galactosidases. Previously, it was suggested that the plant enzymes that cluster with animals might have been transferred to plants by horizontal gene transfer [12], but the fact that the bryophyte, which is thought to have diverged from the vascular plants early in plant evolution, has this type of β-galactosidase and the broad distribution of organisms with this type of β-galactosidase suggest that plants maintained a copy of this gene when plants and animals diverged. Perhaps the more relevant question is how the plant specific Bgal ancestor came to be, and how the C-terminal Gal-lectin-like domain was acquired by this lineage. It appears that the fungal-type and plant-type lineages may have diverged from the animal-type β-galactosidases early in GH35 evolution, but plants retained the animal-type β-galactosidase, as well.

Within the plant-type β-galactosidases, OsBgals can be divided into three distinct groups (a, b and c in Figure 1). The major group (group a) contains 5 clusters with total 7 OsBgal members. The cluster a1 contains 4 rice genes, including OsBgal1, OsBgal2, OsBgal3, and OsBgal7, along with 6 AtBGALs, the *Asparagus officinalis *Bgal, and 4 tomato and 5 chickpea Bgals that are not shown, some of which contain C-terminal galactose-binding lectin-like domains and some of which do not. Another cluster, a2, contains OsBgal13, AtBGAL9 and two putative *P. patens *Bgals (EDQ59880 and EDQ81397), which suggests this cluster is of ancient origin. Two other putative *P. patens *Bgals fall in cluster a3, which appears to be closely related to cluster a2. OsBgal8 groups with AtBGAL8 and RsBgal1 of radish, which specifically hydrolyzes the β-(1→3)- and β-(1→6)-linked oligogalactans of AGPs [18], in subsgroup a4. OsBgal4 groups with AtBGAL10 in cluster a5. The second biggest group, b, contains 4 rice Bgals with high amino acid sequence similarity, namely OsBgal5, OsBgal12, OsBgal14, and OsBgal15. These isozymes have nearly the same protein lengths and gene structures, as described below. Within group c, OsBgal10 and OsBgal11 (cluster c1) are closely related, while OsBgal6 (cluster c2) is less clearly associated, though both distance-based and maximum parsimony trees back this model.

### Chromosomal locations of OsBgals

**Figure 2:**
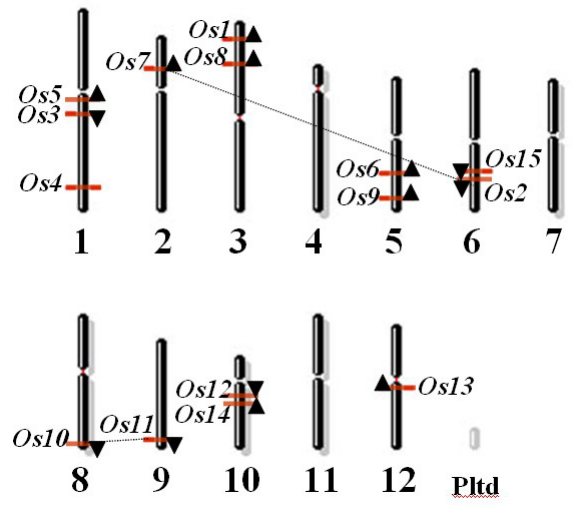
**Distribution and direction of *OsBgal *genes on rice chromosomes.** The chromosome numbers are indicated at the bottom of each bar. Genes lying on duplicated segments of the genome have been joined by dashed lines. Os is short for OsBgals. The gene map was generated with the NCBI map viewer.

As shown in the map of their chromosomal locations and directions of transcription in Figure 2, the *OsBgals *are distributed over all chromosomes, except chromosomes 4, 7 and 11. Three *OsBgals *are located on chromosome 1, while 2 *OsBgal *each are found on chromosomes 3, 5, 6, and 10, and one *OsBgal *each is present on chromosomes 2, 8, 9, and 12. Similar to other gene families, *OsBgals *appear to have undergone gene duplication, as the rice genome has undergone genome-wide duplication events, including polyploidy, which promote the amplification of gene family members. Segmental duplication analysis identified four *OsBgals *located on the duplicated segmental regions in rice chromosomes. *OsBgal7 *(Rice genome project locus Os02g0219200) and *OsBgal2 *(Os06g0573600) are located on a chromosomal segment duplicated between chromosomes 2 and 6, while *OsBgal10 *(Os08g0549200) and *OsBgal11 *(Os09g0539200) are located on segments duplicated between chromosomes 8 and 9. These duplications are supported by the close phylogenetic relationships of these genes. Therefore, chromosomal segment duplication appears to have played a role in multiplication of *OsBgals *within the rice genome. In contrast, *OsBgal12 *and *OsBgal14*, which have high sequence similarity, are located close together on the same chromosome. Thus, these two genes may have diverged by tandem duplication.

### Exon-intron organization and primary gene structure analysis

Automated annotations for all *OsBgal *genes were available in the public databases, except for *OsBgal6*, *OsBgal14 *and *OsBgal15*. However, we confirmed and corrected exon-intron organization manually by comparing the corresponding full length cDNA and EST sequences, which were either already available in publicly accessible databases or were obtained during this study, with genomic sequences.

**Figure 3:**
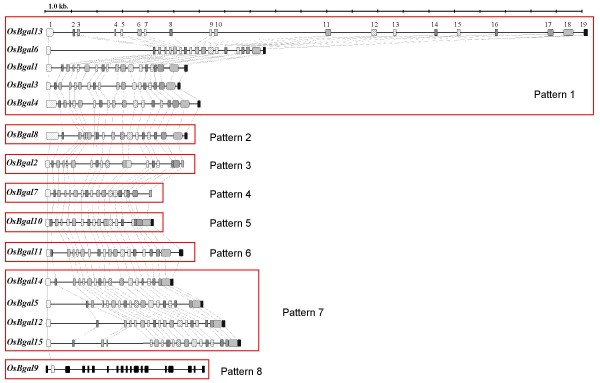
**The exon-intron organization of *OsBgal *genes.** Exons are shown as boxes with corresponding exons having the same pattern. Introns are represented by lines. Groupings of the genes with the same splice pattern are enclosed by red boxes.

Gene structure comparisons showed eight different splicing patterns. The sizes of the majority of the coding exons are conserved, but some appear to have had intron-loss events (Figure 3). The pattern with the highest number of exons, found in *OsBgal1, OsBgal3, OsBgal4*, *OsBgal6 and OsBgal13*, contains 19 exons with 18 introns, with exons 1 through 10 encoding the GH35 domain, and exons 18 and 19 encoding the Gal-lectin-like domain. Ahn et al [12] observed this same pattern in *A. thaliana Bgals *and surmised from parsimony that it is likely to be the ancestral gene pattern and other patterns were derived from it, mainly by intron loss. The presence of this pattern in diverse rice genes and in a *P. patens *gene (EDQ59880) supports this conclusion. The second most similar pattern is 18 exons separated by 17 introns with the loss of intron 1, which occurred in *OsBgal11*. A pattern of two-intron loss was found in *OsBgal5, OsBgal12*, *OsBgal14*, and *OsBgal15*, which contain 17 exons with the loss of introns 17 and 18.

The *OsBgal2*, *OsBgal7 *and *OsBgal9 *genes all lack the galactose-binding, lectin-like domain at the C-terminus, however, their splicing patterns were different. *OsBgal2 *and *OsBgal7 *have patterns similar to other rice GH35 genes and retain the 5' end of exon 18, but *OsBgal2 *contains 16 exons with the loss of introns 11 and 16, while intron 15 has been lost in *OsBgal7*, leaving 17 exons (Figure 3). In contrast, *OsBgal9 *appeared to be distantly diverged from other rice GH35 members since its exon/intron organization does not correspond to the other *OsBgals*, except that an intron is found in the same position as intron 1 in the other genes.

Although the pattern and lengths of exons in rice GH35 genes are similar, *OsBgal13 *spans approximately 20.8 kb, because it contains over three-fold longer introns than the other genes in the family. Long terminal repeat (LTR)-retrotransposons elements and other repeated sequences were found within *OsBgal13 *gene introns 1, 2, 8, 9, 10, 13, 14 and 16, which contain one GC rice region, three AT rich regions and eight transposable elements (Table 3). Likewise, *OsBgal6*, the second largest *OsBgal *gene due to its long (4 kb) intron 1, had one AT rich region and seven transposable elements, all of which were located within intron 1. In *OsBgal *genes of normal length, such as *OsBgal1*, no transposable elements were observed. These results may reflect the observation that rice genome expansion is forced by transposable element amplification [40].

**Table 3:** Identification of LTR-retrotransposon elements and DNA sequences for interspersed repeats and low complexity DNA sequences within *OsBgal6 *and *OsBgal13*.

Genes	LTR-retrotransposon elements and DNA sequences for interspersed repeats						
	
	intron 1	intron 3	intron 8	intron 10	intron 13	intron 14	intron 16
*OsBgal6*	Stowaway41_OS, OLO24C, OLO24B, RPO_OS, AT rich Low Complexity, AMYLTP, TA rich Low Complexity, OSTE15						
*OsBgal13*	GC rich Low complexity	AT rich Low complexity	MUDRN2	OSTE28, OSTE33, CRM-I_OS LTR/Gypsy,	OSLINE1-5, AT rich Low complexity	AT rich Low complexity	RdSpm875A_3.1, ID-2 DNA/Tourist

RepeatMasker  was used to identify these sequences.

Comparison of gene splice patterns and phylogenetic relationships between plant-type Bgals from rice, *A. thaliana*, and *P. patens *reveals that, although the ancestral gene pattern is the same, no other splice patterns are shared within a phylogenetic lineage. Therefore, though most introns appear to have been inserted in the common ancestor gene for this lineage (an extra exon was inserted in *P. patens *gene EDQ878340), intron losses appear to have occurred independently in rice, *Arabidopsis *and *P. patens*. The same may be true for the C-terminal domain, which appears to have been lost only once in rice (in the putative ancestor of *OsBgal2 *and *OsBgal7*), though it appears to have been lost at least 4 times in *Arabidopsis *GH35 *Bgals *[12].

### Analysis of expression by RT-PCR

**Figure 4:**
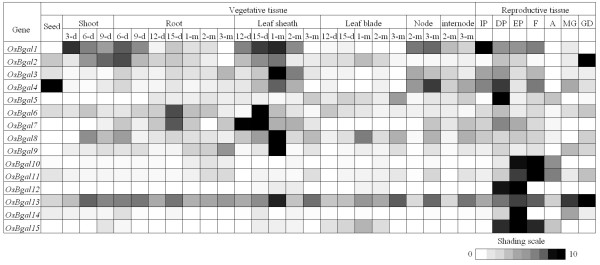
**Relative expression levels of 15 *OsBgal *genes in different tissues determined by semiquantitative RT-PCR.** Signals were quantified and normalized to the expression of *β-Actin*, and the highest observed expression level of each gene was designated as 10 (black), with other tissues expression set relative to this maximal level. See Additional file 1 for actual values and standard deviations. IP, initiating panicle; DP, differentiating panicle; EP, exerting panicle; F, flower; A, Anther; MG, milk grain; GD, grain during dry down; d, day(s); m, month(s).

*OsBgal *transcript expression in various tissues and stages of growth was analyzed by RT-PCR with primers specific to the 3'UTR of each gene to gain further insight into possible functions. The relative expression levels derived from normalization with the *UBQ6 *polyubiquitin gene gave the same patterns as those shown for normalization with the *β-Actin *gene in Figure 4 and Additional file 1, indicating the patterns likely reflect the expression of the *OsBgal *genes, rather than that of the control.

The rice tissues/organs and developmental stages chosen for RT-PCR analysis are shown in Figure 4. All 15 *OsBgal *genes were found to be expressed with different but overlapping expression patterns, confirming that all genes are active. These relative expression levels were used to define genes as having expression specific to a given tissue, if the relative transcript levels of the gene at that stage are significantly higher (color scale above 7, where 10 is the level in the tissue with the highest expression of each gene) over the levels at all other stages. The expression profiles revealed that at least 9 genes were highly expressed in one of the stages of vegetative tissues and 10 were highly expressed in reproductive tissues. Only *OsBgal13 *appears to be expressed in nearly every organ and developmental stage, though at different levels (Figure 4).

Nine *OsBgals *(*OsBgal1, OsBgal2, OsBgal3, OsBgal4, OsBgal6, OsBgal7, OsBgal8 and OsBgal9*) displayed their highest transcript levels in young and early-mature stages of shoot, root, and leaf sheath. Among these genes, *OsBgal6, OsBgal7, OsBgal8 and OsBgal9 *showed highest intensity in leaf sheath at different times, especially, 15-day and 1-month old, which are both fast cell elongation stages. The high accumulation of OsBgals in this stage may act in sugar remodeling, since the carbohydrate remobilized from the leaf sheath and culm to grain can contribute as much as 38% to rice yield [41]. In contrast, low levels of most gene transcripts were detected in leaf blade, except for *OsBgal13 *transcripts, which were detected throughout the growth of the blade.

Six genes, *OsBgal5, OsBgal10, OsBgal11, OsBgal12, OsBgal14*, and *OsBgal15 *appear to be expressed primarily in reproductive tissues. The relative transcript levels for these genes were at least five times higher in reproductive tissues than in vegetative tissues (Figure 4). In addition, no expression was detected at the initiating panicle stage, but it was high in later panicle development and flowering stages. Only *OsBgal10, OsBgal11 *and *OsBgal15 *transcripts were found to accumulate in anther. These genes appear to have diverged from two ancestor genes, since *OsBgal5*, *OsBgal12*, *OsBgal14*, and *OsBgal15 *have sequences and gene structures that are similar to each other, as do *OsBgal10 *and *OsBgal11 *(Figures 1 and 3). Interestingly, *AtBGAL7 *and *AtBGAL15*, which fall into the group b with *OsBgal5*, *OsBgal12*, *OsBgal14*, and *OsBgal15 *(Figure 1), were reported to function in the early stages of microspore and pollen development [31]. Likewise, *AtBGAL11 *and *AtBGAL13*, which fall in cluster c1 with *OsBgal10 *and *OsBgal11 *(Figures 1), show maximum expression in mature pollen [31]. So, related rice and *Arabidopsis *Bgals function in reproductive tissues, suggesting their roles may be somewhat conserved in the two plants.

Only the *OsBgal4 *transcript was expressed abundantly in seed at the imbibition stage, while *OsBgal2 *and *OsBgal13 *were also present at low levels. Five *OsBgals *were expressed in milk grain and six in grain during dry down. A high abundance of *OsBgal2 *and *OsBgal13 *transcripts was observed in grain during dry down. This evidence implies that they may function in grain development and senescence or are stored for roles in germination, such as cell wall remodeling. *OsBgal1*, *OsBgal2, OsBgal6, OsBgal8*, and *OsBgal13 *transcripts were also found in shoots and roots of seedlings after germination. Chantarangsee et al. [38] reported β-galactosidase activity in rice seeds, roots and shoots at 0–7 days after seed-soaking, and detected OsBgal1 and OsBgal2 proteins in embryos and seedling roots and shoots. In barley (*Hordeum vulgare*), Giannakouros and colleagues [17] were able to separate 4 isozymes of β-galactosidase from germinated grain. Thus, Bgals are present and may play roles in the growth and emergence of root and shoot primordia from the hull.

To complement the RT-PCR expression profiles of the *OsBals*, ESTs retrieved from the UniGene database (UniGene December, 2007, NCBI) were compared with our findings. Full-length or partial cDNA and ESTs for most *OsBgals *are available in the database, except *OsBgal6*, for which only one EST from unspecified tissues is available, and *OsBgal15*, for which no corresponding ESTs or cDNA are available (Table 1). However, the other *OsBgal *genes had cDNA and ESTs present in the database, with numbers of ESTs that varied from 1 to 253.

The *OsBgal8 *(Unigene cluster Os.22360, locus *Os03g0255100*) has the highest number of ESTs in the database: 253 ESTs from various tissues, including flower, stem, leaf, panicle, root, callus, seed, whole plant and unspecified tissues. The Unigene profiles indicate relatively abundant accumulation in flower, leaf, panicle, root, seed and stem with 761, 133, 120, 190, 216 and 228 transcripts per million (TPM), respectively (Table 1). The Unigene profile is somewhat similar to the RT-PCR pattern of *OsBgal8*, in which the transcripts accumulated significantly throughout rice plant, including in seed during dry-down, but not in seedling and milk grain.

*OsBgal10 *(Os.18310), *OsBgal11*, *OsBgal12 *(Os.46702) and *OsBgal14 *(Os.22528) expression data included mainly flower and panicle clones, or had relatively high expression in these tissues, which correlates to our RT-PCR analysis. For instance,*OsBgal11 *shows 249 TPM in flower and 158 TPM in panicle, while *OsBgal14 *shows 358 TPM in flower (Table 1).

The EST and cDNA clones for the remaining *OsBgals *had diverse origins, which is consistent with the evaluation of *OsBgals *by RT-PCR, though the frequencies of expression derived from these two forms of expression data were not exactly the same. Therefore, Unigene database analysis could be used for an initial guideline of gene expression, though the expression patterns based on ESTs are fragmentary, since the tissues are not equally or completely represented and the source tissue annotation of many EST libraries is unclear or incomplete. Microarray experiments, such as that by Ma et al. [42], who assessed expression of 37,132 non cross-hybridizing gene models in rice seedling shoots, tillering-stage shoots and roots, heading and filling stages of panicles and suspension cells, offer a more comprehensive picture of gene expression within a tissue. Although the tissue coverage in public microarray databases is as yet limited, the GEO expression data in Table 1 shows that OsBgal expression levels may be affected by many treatments.

### Rice β-galactosidase cDNAs sequencing and identification of alternative splicing

Although the expression of all *OsBgals *can be observed by RT-PCR and most have corresponding cDNAs and ESTs in the database, complete full-length cDNA clones were not available for *OsBgal6*, *OsBgal11 *and *OsBgal15*. Therefore, to confirm the mRNA sequence and gene structure, these genes were amplified by PCR and sequenced. The accession numbers for these sequences are listed in Table 1.

**Figure 5:**
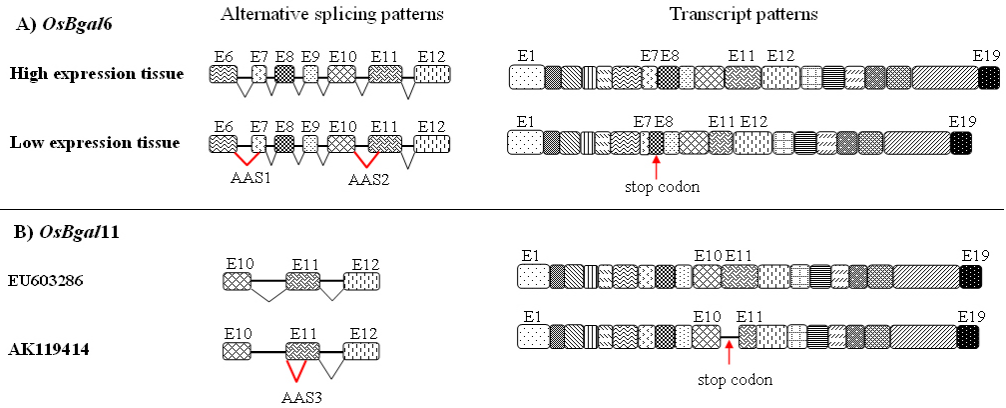
**Alternative splicing of *OsBgal6 *(A) and *OsBgal11 *(B).** The high expression tissue for *OsBgal6 *is 15-day-old rice leaf sheath, while the low expression tissue is exerting panicle. For OsBgl11, the splicing pattern of the cDNA amplified from exerting panicle of KDML105 indica rice (Genbank accession EU603286) is shown above, while the database sequence from Nipponbare japonica rice (AK119414) is shown below. Abbreviations include AAS: alternative acceptor site, E: Exon. AAS1: alternative acceptor site (CAG) located 80 nt downstream of the functional acceptor site; AAS2: alternative acceptor site (CAG) located 20 nt downstream of the functional acceptor site; AAS3: intron retention and alternative splice site within exon 11. The data for *OsBgal1 *and *OsBgal2 *were previously reported by Chantarangsee et al. [38].

The full-length cDNA of *OsBgal*6 was amplified by a set of primers corresponding to the transcribed locus XM_475258. The cDNA was isolated from 15-day leaf sheaths, a tissue with abundant expression, and exerting panicles, a tissue with poor expression, based on RT-PCR expression analysis. Sequencing of the cDNA from the highly expressing tissue revealed an open reading frame (ORF) of 2,436 nucleotides encoding a protein of 812 amino acids (Genbank Accession EU602310), which confirmed the our predicted β-*galactosidase *sequence from chromosome 5 (Genbank accession no. AC135419 and AC135429). A search through the dbEST database of *O. sativa *found 99% identity with a 520 bp EST from fertile panicle (CK048087). On the contrary, the Os *Bgal*6 cDNA amplified from exerting panicle, where expression is low, contains only 2,338 bp (Genbank Accession EU603285) which is 101 bp shorter than the cDNA from high expression tissues. The open reading frame beginning at the initiation codon of this transcript covers only exons 1 to 7 and encodes only 244 amino acids. Alternative splicing of this transcript occurred to utilize an alternative acceptor site (CAG) 80 nt downstream of functional acceptor site for intron 7, which introduced a deletion of 27 amino acid and a frameshift that leads to an in-frame stop codon (Figure 5). A second missplicing of this gene occurred at the intron 10/exon 11 junction, which again used an alternate acceptor site (CAG) located 20 nt downstream of the regular acceptor site. So, alternative splicing is likely to be one of the gene regulation mechanisms, since the transcript would be subject to nonsense mediated decay (nmd) [43] and the protein produced would be useless as a β-galactosidase.

*OsBgal11 *(locus Os09g0539200) is annotated as a pseudogene in GenBank, because of its apparent inability to produce full-length protein due to a frameshift and subsequent stop codon in the AK119414 cDNA. We hypothesized that such phenomenon probably resulted from missplicing, as seen in *OsBgal6*. A 1343-nucleotide fragment from the 3' half of the *OsBgal11 *cDNA was amplified from exerting panicle (Genbank Accession EU603286), in which its expression is high based on RT-PCR. This cDNA shares 98% identity with the AK119414 cDNA, but had a substitution of 75 nucleotides in place of 95 nucleotides at positions 1282 to 1377 of AK119414. These substituted sequences allow the full-length translation of the predicted 839 amino acids of OsBgal11, whereas only 426 amino acids are translated from AK119414. Comparison with the genomic sequence and our amplified cDNA sequence indicates that the AK119414 transcript results from retention of intron 10, which extends exon 10 by 101 nt, thereby introducing an in-frame stop codon. In addition, an alternative donor site located at the functional acceptor site of intron 10 and the first nucleotide of the functional exon 11 produces an alternate intron 10 with the acceptor site located 79 nt downstream, within the functional exon 11, thereby reducing the size of exon 11. The shortened (456-residue) protein, includes all of the catalytic domain, based on the *Penicillium *sp. β-galactosidase structure [44], so it is possible that it could still function as a Bgal.

No experimentally determined cDNA or ESTs corresponding to *OsBgal15 *were available in public databases, despite the fact that our RT-PCR demonstrated its expression. Therefore, the full-length *OsBgal15 *cDNA was amplified, cloned and sequenced. The *OsBgal15 *cDNA sequence (Genbank Accession EU051629) contains a 2487-nucleotide ORF, which encodes 829 amino acids. The encoded protein shares over 99% identity with a predicted β-galactosidase of *Oryza sativa *(japonica cultivar-group) (accession no. AP004733), except that 7 extra amino acid residues were inserted at residue 293, compared to the predicted sequence from AP004733. Therefore, the correct splice donor site of exon 8 is located 21 nucleotides downstream of that predicted for the mRNA of AP004733.

The alternative splicing observed in this study introduces in-frame stop codons into the mRNA of *OsBgal6 *in the low expression tissue, but not for the high expression tissues, while it can also introduce a premature stop codon in *OsBgal11*, based on the AK119414 cDNA sequence. In vertebrates, there are a number of well-known alternatively splicing genes, for instance the fibronectin gene, which can generate over 20 different proteins, some of which have different patterns of localization and slightly different functions in human cells [45]. Until relatively recently, there were very few examples of alternatively spliced genes in higher plants and still fewer examples known to generate functionally distinct proteins. Generally, alternative splicing yields two polypeptides of different size that are identical, except for the presence of a number of additional amino acids at the C-terminus of the larger isoform, eg., ribulose-1,5-bisphosphate carboxylase/oxygenase (Rubisco) activase [46]; diacylglycerol kinase in tomato [47]; and chloroplast ascorbate peroxidase in spinach [48]. Missplicing events were observed in maize (*Zea mays*) *Viviparous 1 *(*Vp1*) transcripts, the majority of which are spliced incorrectly and contain insertions of intron sequences or deletions of coding region, so that they do not encode full-length proteins [49]. Although alternative splicing is a widespread process used in higher eukaryotes to regulate gene expression and functional diversification of proteins [50,51], the putative control of tissue-specific expression by missplicing we observed has not been well characterized in rice.

### Preliminary Characterization of OsBgal13

OsBgal13 is in cluster a2 of group a of the phyloenetic tree in Figure 1, along with AtBGAL9 and two *P. patens *Bgals. This and its general expression pattern suggest it may have a conserved maintenance function in plants. To assess what its function might be, OsBgal13 was expressed as a thioredoxin fusion protein in *Escherichia coli *strain OrigamiB (DE3), which we previously found to have little background β-galactosidase acitivity [38]. The protein was purified by immobilized metal affinity chromatography on nickel resin, and most of the OsBgal13 activity eluted in 5–20 mM imidazole. Further purification was achieved by gel filtration chromatography and the activity of the resulting protein was assessed. As shown in Table 4, among *p*-nitrophenol (*p*NP) glycosides, the OsBgal13 enzyme had highest activity toward *p*NP-β-D-galactoside (*p*NPGal), but also had 30% of this activity toward *p*NP-α-L-arabinoside (*p*NPAra), and low activity toward *p*NP-β-D-fucoside and *p*NP-β-D-mannoside, but no detectable activity toward *p*NP-β-D-glucoside and *p*NP-β-D-xyloside and negligible activity toward *p*NP-α-D-galactoside. OsBgal13 also hydrolyzed β-(1→3)-, β-(1→4)- and β-(1→6)-linked galactobiose and galactotriose, but no release of galactose or arabinose could be detected when the protein was incubated for 24 h with rice seedling alcohol insoluble residue (AIR), larchwood arabinogalactan, galactan, apple pectin, citrus pectin or oat xylan. So, despite the intriguing observation that OsBgal13 can hydrolyze both β-D-galactosyl residues and α-L-arabinosyl residues, components of type I and II arabinogalactans and xyloglucan and glucuronoarabinoxylan side chains [52], only short galacto-oligosaccharides can be identified as possible natural substrates to date.

**Table 4:** Hydrolysis of *p*-nitrophenyl glycosides, oligosaccharides and polysaccharides by OsBgal13.

Substrate	Activity
*p*NP-glycosides	(Percent Relative Activity)
*p*NP-β-D-galactopyranoside	100
*p*NP-β-D-glucopyranoside	N.D.
*p*NP-β-D-fucopyranoside	7.4
*p*NP-β-D-mannopyranoside	4.5
*p*NP-β-D-xylopyranoside	N.D.
*p*NP-β-L-arabinopyranoside	30
*p*NP-β-D-galactopyranoside	1.5

Oligosaccharides	(estimated relative activity)
β-1,3-galactobiose	+++
β-1,3-galactotriose	++
β-1,4-galactobiose	++ *
β-1,4-galactotriose	++ *
β-1,6-galactobiose	+++
β-1,6-galactotriose	++
Arabinogalactan, xylan from oat spelts, birchwood xylan, polygalacturonic acid, apple pectin, citric pectin, galactan	N.D.

N.D. means not detectable. * For β-1,4-galactotriose and β-1,4-galactobiose transglycosylation was observed as much or more than hydrolysis.

### Possible Functions of Other Isozymes

The activity of mammal β-galactosidase specifically releases terminal β-galactosyl residues from glycosaminoglycans and the glycolipid GM1 ganglioside. Similarly, the digestion of the galactolipid monogalactosyldiacylglyerol by vacuolar and chloroplast β-galactosidases was reported in wheat [53]. Likewise, β-galacosidase of tomato was reported to act in galactolipid turnover and degradation, which occurs in chloroplasts and chromoplasts during tomato fruit development [54,55]. However, relatively little is known about metabolism of other glycolipids, glycopoteins and proteoglycans involving plant β-galactosidases, though it is well characterized in mammals. The fact that OsBgal9 is similar to animal β-galactosidases and is predicted to localize to a lysosome-like vacuole, suggests it may play a similar role in rice.

Among the plant-like OsBgals, OsBgal10 and OsBgal11 are also predicted to localize to lysosome-like vacuoles, so they could also play a role similar to animal-type β-galactosidases. However, these closely related isozymes have reproductive tissue specific expression, as are the isozymes of cluster b, OsBgal5, OsBgal12, OsBgal14 and OsBgal15. Similarly, AtBGAL11 and AtBGAL13, which clustered in group c1 with OsBgal10 and OsBgal11, and AtBGAL7 and AtBGAL15, which clustered in group b, were found to be expressed in flower [12] and pollen [31]. It may be that the ancestral genes for these enzymes developed reproductive-tissue specific roles before the ancestors of monocots and dicots diverged. If these functions are conserved, these isozymes may have similar roles in the two plants, though these functions remain to be determined.

Among the other plant-like genes, it is likely many of them act in cell wall metabolism, but these roles are yet to be discerned. OsBgal1 was shown to hydrolyze β-(1→3)-, β-(1→4)- and β-(1→6)-linked oligosaccharides and, at a low level, arabinogalactan and be expressed in a range of vegetative and reproductive tissues [38], so it may have a general role on similar substrates in the cell wall. OsBgal2, OsBgal3 and OsBgal7 are closely related to it, as are 4 tomato Bgals involved in fruit ripening [13] and an asparagus Bgal thought to act in senescence [56], so a cell wall function seems likely for these other cluster c1 isozymes as well, though such a role must be proven. Kaneko and Kobayashi [37] showed that OsBgal8, which is secreted from rice tissue culture cells, could release galactose from galactoxyloglucans and pectic galactans. So, a role in cell wall metabolism is quite likely for this group a isozyme.

The role of OsBgal6 remains obscure, as no closely related enzyme has been characterized, however it seems to be specific to vegetative tissues of the young plant. The fact that the transcript found in panicle was misspliced to prevent production of active enzyme is intriguing, but the functional implications of the alternative splicing of both OsBgal6 and OsBgal11 remain to be clarified.

## Conclusion

Fifteen rice glycosyl hydrolase family 35 genes encoding putative β-galactosidases were identified in this study. This number is similar to the 17 seen in *Arabidopsis *and these plants appear to contain 9 common gene lineages present in their ancestors before they diverged [12]. OsBgal9 was clustered into the same group as Bgals from animal species, such as *H. sapiens *and *D. melanogaster *in the phylogenetic tree, while the other rice BGals fall in a nearly plant-specific subfamily of Bgals, most of which contain a C-terminal lectin-like domain. The presence of both types of β-galactosidase in the moss *P. patens*, a nonvascular plant, suggests that both types of genes were present early in plant evolution. Within the plant-type Bgals, group a (Figure 1), contains 7 OsBgals and many Bgals that have been characterized from other plants (for example, 9 of *Arabidopsis*, 4 of *C. arietinum *and 3 of *L. esculentum*), including some with and some without C-terminal galactose-binding-lectin-like domains. Many of these proteins, including OsBgal8 [37], appear to act on the cell-wall-derived substrates. The isozymes of phylogenetic groups b and c1, OsBgal5, OsBgal10, OsBgal11, OsBgal14, and OsBgal15, appear to have reproductive-tissue specific functions, while OsBgal6 (group c2) is predominantly in young leaves and roots. *OsBgal6 *appeared to undergo alternative splicing to prevent its production in panicle, and alternative splicing was also found for *OsBgal11*. Understanding the functions of this gene family and significance of alternative splicing in this will require further functional investigation.

## Methods

### Sequence data and database search

To find all GH35 genes in rice, tBLASTn searches [57] were performed in the National Center for Biotechnology Information (NCBI) Genbank nr, indica rice genome, and expressed sequence tags (dbEST) databases with *OsBgal1 *(AK102192) as the query. The retrieved *OsBgal *genes were used to identify their Unigene cluster, Gene Locus, conserved domains, gene position on the 12 *Oryza sativa *(rice) chromosomes, homolog genes, EST-based expression profiles and GEO profile in the NCBI databases (please see Availability & requirements for more information). The number and positions of exons and introns for each individual gene were determined by manually comparing the cDNA and predicted cDNA sequences with their corresponding genomic DNA sequences. Homologous proteins from other organisms were retrieved by links from the CAZY website (please see Availability & requirements for more information) or by BLASTp searches at NCBI. Putative signal peptides were predicted with the SignalP program (please see Availability & requirements for more information) [58];  and putative N-glycosylation sites were identified with the NetNGlyc [59]; (please see Availability & requirements for more information) and manually inspected to remove NPS/T sites.

Chromosomal locations of genes were identified on and the map drawn with the NCBI map viewer (please see Availability & requirements for more information). Segmental duplication analysis was done with DAGchainer [60] and the TIGR rice segmental duplication database (please see Availability & requirements for more information) with the maximum length distance permitted between collinear gene pairs set to be 500 kb. LTR-retrotransposon elements, interspersed repeats and low complexity DNA sequences were identified with RepeatMasker (please see Availability & requirements for more information).

### Construction of phylogenetic trees

The multiple sequence alignment of β-galactosidase protein sequences from rice and other organisms was made with ClustalW [61] and manually adjusted and edited to remove unconserved N- and C-terminal regions with Genedoc (please see Availability & requirements for more information) [62]. Phylogenetic trees were constructed by the neighbor-joining facility in ClustalX 1.83 [63] and the branching order verified for maximum parsimony with the Protpars program in the PHYLIP software suite [64].

### Plant material

Indica rice (*Oryza sativa *L., cv. KDML 105) seeds were soaked under sterile conditions on tissue paper moistened with sterile distilled water at 28°C until germination. Germinated rice was moved to soil-filled plastic pots and grown until 15 days, then moved to large clay pots. Samples were collected every 3 days from seeding until 15 days after seeding, then sampling was continued every month. To attain more materials at the flowering stage, samples were collected from KDML105 fields at the Pathum thani Rice Research Center in November, 2005. The samples were immediately frozen in liquid nitrogen and stored at -80°C until use.

### Semi-quantitative reverse transcription-polymerase chain reaction (qRT-PCR)

Total RNA was isolated from various tissues: germinated seed, root, shoot, leaf blade, leaf sheath, node, internode, initiating panicle, developing panicle, emerging panicle, anther, flower, milk grain, and grain during dry down by the following procedure. Tissues (100 mg) were ground to powder under liquid nitrogen, then RNA was extracted using RNeasy Plant Mini (QIAGEN GmbH, Hilden, Germany) or Sigma Spectrum RNA extraction (Sigma-Aldrich, St Louis, USA) kits. For starchy tissues: germinated seed and grain during dry down, RNA extractions were first done with Trizol reagent (Invitrogen, Carlsbad, CA, USA [65]), and the RNA further purified from the extracts by the spin column procedure of the above RNA extraction kits. The RNA was quantified in a spectrophotometer at 260 nm.

For RT-PCR, approximately 5 μg of total RNA was treated with RQ1 RNase-free DNase I (Promega Corporation, Madison, WI, USA). First stand cDNA was synthesized from RNA template primed with Oligo (dT)_20 _with the SuperScript III First-Strand Synthesis System for RT-PCR (Invitrogen). The gene-specific primers used for semi-quantitative PCR were designed from the 3'UTR or 3' coding sequence of each β-galactosidase gene (Table 5). The constitutively expressed *β-Actin *and *Ubiquitin-6 *genes were used for normalization. The PCR (10 μl total volume) was done with a 10 ng or 100 ng aliquot of the first stand cDNA as template and 0.5 units of *Taq *DNA polymerase (Promega). The relative expression abundances were obtained by dividing the total densimetric intensities measured on a Fluor-S gel documentation system with Quantity 1 software (Bio-RAD, Hercules, CA, USA) for each gene by those for the control gene. All samples were assayed 3 times in separate reactions to give means and standard deviations for the relative abundances.

**Table 5:** Sequences of primers used for semi-quantitative RT-PCR analysis of β-galactosidase gene expression.

Gene name	GenBank ID	Sense primer	Antisense primer	Annealing Tm (°C)
Actin	AK100267	ACTCTGGTGATGGTGTCAGCC	GTCAGCAATGCCAGGGAACATA	54
*UBQ6*	XM464194	TCCTCCGTCTCAGGGGAG	CTTGCCAGCGAAGATCAGAC	54
*OsBgal1*	AK102192	CAAAGCACACAGAAAGCGAT	TGCTCACCGCACAATCAACGA	54
*OsBgal2*	AK102756	AGGAAAGTGGGGGCGTATAG	TCCCATTTACAACTCAACGT	56
*OsBgal3*	AK103045	CCAAGGGGCTGTATGTATGGTC	CCTGAGAGAATTCATTCACATACGG	56
*OsBgal4*	AK102715	TCCATCGCTACAGATTCGCTC	TCCAGAAATATCATGACGCGAC	56
*OsBgal5*	AK119447	CTCATCTGCTTGCTTCATC	CTAAAGTTGCCCTTCTCATC	50
*OsBgal6*	CR292731	AGTCTTGCATAGGCAGGAG	TCTGAACGAAGGTATCGCAC	54
*OsBgal7*	AK059059	CCACCATTTGATACAGTCGTCG	TTCCCGAGCAACGCAAAC	54
*OsBgal8*	AK067479	CCTGACAGGTTTGATAGTGCTCG	TGCTTTTCTTCACACAAGTGCATC	54
*OsBgal9*	AK068572	GAAGGATCCAGATTTCACATGC	TGCTGTTCATGTCATCATGTGC	54
*OsBgal10*	AK069066	TCCAAGAGGCCTCCTCGTTC	CTACATATAAAACCATGGACGATGGTG	54
*OsBgal11*	AK119414	GTGCAGGTGAGATGCAAGGTATC	CAAACTGTCTGTCAACCTGTGATGG	54
*OsBgal12*	AK119350	CAACACAGCAAAACCATCT	TTCCACGAAACAAAGTAAAGACA	56
*OsBgal13*	AK065546	CCGAGGAGTCCTCAAAGATTTAGC	GGAAATCTCCTTTGCATTTTTATTCAC	54
*OsBgal14*	AK242960	GTCGAAGGTGGCTTATGACG	AATCGACAGTGCGGTATCTC	54
*OsBgal15*	AP004733	CGTACAAGGCTTTCACAGAAG	CTACGATTACTTGATCACACTC	54

### Amplification of cDNA clones and sequencing

The first strand cDNA reverse-transcribed from RNA of developing and emerging panicle was used as a template to amplify *OsBgal6*, *OsBgal11*, and *OsBgal15 *cDNA. The set of primers for amplification of partial or full-length cDNA of each gene was designed from its genomic sequence. The PCR was catalyzed by *Pfu *polymerase (Promega) with heating at 94°C for 5 min, followed by 30 cycles of 1 min at 94°C, 30 s at the appropriate annealing temperature, (58°C for most primers) and 5 min at 72°C, and a final extension for 7 min at 72°C. The final product was gel purified with the QIAquick Gel Extraction Kit (QIAGEN), then sequenced by automated sequencing at Macrogen Inc. (Seoul, Korea).

Two pairs of primers were used to amplify *OsBgal6 *cDNA: (1), bgal6_startF (TCAGTCAGTAGTCAGACATG) and bgal6_QIENEY_R (AATGCAGGCTCAATCATCAG); and (2), bgal6_QIENEY_F (GATGATTGAGCCTGCATTTG) and bgal6_stopR (AGTTTCCTGTGTTGCATCAC). The full-length sequence of *OsBgal6 *was determined with the above primer sets and additional primers, including bgal6_1163r (GGTGTGTTATGCTGATCGAAG), bgal6_1650f (GGATTCTGGCGCCTACATG), bgal6_1143F (CTTCGATCAGCATAACACACC), and bgal6_1671r (CCATGTAGGCG CCAGAATC).

The oligonucleotide primers used for amplification of a partial *OsBgal11 *cDNA were: bgal11_Seq_r1 (TCATCGCATGTGCAGTG) and bgal11_STOP_R (CCTTCTTCCTAAGCCGCCTG).

The *Osbgal15 *cDNA was amplified with 2 pairs of primers. First, the bgal15_START_F (CGCGTGCCGGCGATGAAG) and bgal15_QIENE_Rev (CTCGTTTTCAATCTGTGCCAG) primers were used to amplify a 5' cDNA fragment, and the bgal15_QIENEY_F (CTGGCACAGATTGAAAACGAG) and bgal15_STOP_R (TATCAACATGAAGCCTGAACGGTG) primers were used to amplify an overlapping 3' cDNA fragment. The products of these first two PCR were mixed and a sequential PCR was performed with the bgal15_START_F and bgal15_STOP_R primers. The full-length CDS cDNA was cloned into the *EcoR*Isite of pBluescript KS(+) by standard methods [66]. The recombinant plasmid was sequenced with the T7, M13, bgal15QIENE_F, bgal15QIENE_REV, bgal15_1138_F (ACTCATCTTCTGCCTGCTTG), and Bgal15_1797_R (TACGGTGCCATTGTTG TTG) primers.

### Expression of OsBgal13

A cDNA encoding the predicted mature OsBgal13 protein was amplified from the full-length cDNA clone of Genbank Accession AK065546[67], obtained from the Rice Genome Resource Center, Tsukuba, Japan. The plasmid clone was used as template for amplification with the bgal13matN (CACCGCCGCCGCGTATGC) and bgal13stopR (GATCACATCTCACCGCGAGGCTC) primers and *Pfu *polymerase (Promega), according the supplier's instructions. The PCR product was gel-purified and cloned into the pENTR/D-TOPO plasmid (Invitrogen), according to the supplier's instructions, and sequenced. The cDNA was transferred into the pET32/DEST vector [68] by an LR clonase reaction (Invitrogen). The thioredoxin fusion protein was expressed from this plasmid and expressed as described for OsBgal1 by Chantarangsee et al. [38]. The protein was extracted and purified on Ni-NTA IMAC resin (QIAGEN), as with OsBgal1 [38], except that 0.1 mg/mL soybean trypsin inhibitor was added to the extraction buffer, and the protein was eluted from the IMAC column with 5–20 mM imidazole. The OsBgal13 protein was further purified by gel-filtration on a Superdex S200 column. The purified OsBgal13 was tested for glycone specificity by incubation of enzyme with 1 mM *p*-nitrophenyl (*p*NP) glycosides for 30 min at 30°C, followed by stopping the reaction with 2 volumes 0.4 M Na_2_CO_3 _and measuring the 405 nm absorbance on a microtiter plate. Action on galactose-containing oligosaccharides and polysaccharides was determined by incubating with 1 mM oligosaccharide or 0.5% polysaccharide at 30°C 24 h, then separating the products on TLC and detecting carbohydrates, as previously described [38].

## Availability & requirements

NCBI databases: 

CAZY website: 

SignalP program: 

NetNGlyc: 

NCBI map viewer: 

PHYLIP: 

TIGR rice segmental duplication database: 

 RepeatMasker: 

Genedoc: 

## Authors' contributions

WT performed rice plant cultivation, RT-PCR analysis, cDNA cloning and sequencing, and drafted the manuscript. WT, JK–C and MC carried out gene structural analysis. WT and JK–C participated in data analysis, and gene and protein analysis. JM expressed, purified and characterized the recombinant OsBgal13 protein. JK–C organized and directed the project and helped to draft the manuscript. All authors read and approved the final manuscript.

## Competing interests

The authors declare that they have no competing interests.

## Supplementary Material

Additional file 1 **Relative expression levels of 15 *OsBgal *genes in different tissues determined by semiquantitative RT-PCR**. Signals were quantified and normalized to the expression of β-actin. The values given are means with standard deviations from triplicate experiments. Click here for file
